# General Bill of Mortality for London, from Dec. 1835, to Dec. 15, 1836

**Published:** 1837-07-01

**Authors:** 


### OBITUARY.

### General Bill of Mortality for London, from Dec. 15, 1835, to Dec. 15, 1836.

| *Diseases.* | |
|---|---|
| Abscess | 110 |
| Age & Debility | 2320 |
| Apoplexy | 405 |
| Asthma | 853 |
| Cancer | 123 |
| Childbirth | 172 |
| Cholera | 2 |
| Consumption | 3238 |
| Constipation of the Bowels | 14 |
| Convulsions | 1617 |
| Croup | 169 |
| Dentition, or Teething | 393 |
| Diabetes | 1 |
| Diarrhœa | 21 |
| Dropsy | 847 |
| on the Brain | 578 |
| on the Chest | 54 |
| Dysentery | 7 |
| Epilepsy | 24 |
| Erysipelas | 75 |
| Fever | 354 |
| (Intermittent or Ague) | 2 |
| (Scarlet) | 261 |
| (Typhus) | 59 |
| Fistula | 6 |
| Gout | 91 |
| Hæmorrhage | 33 |
| Heart, diseased | 155 |
| Hernia | 10 |
| Hooping-cough | 311 |
| Hydrophobia | 1 |
| Indigestion | 10 |
| Inflammation | 1482 |
| Bow. & Stom. | 236 |
| Brain | 165 |
| Lungs & Pleura | 272 |
| Influenza | 5 |
| Insanity | 226 |
| Jaundice | 35 |
| Jaw, locked | 6 |
| Liver, diseased | 246 |
| Measles | 404 |
| Miscarriage | 16 |
| Mortification | 194 |
| Paralysis | 164 |
| Rheumatism | 32 |
| Scrofula | 29 |
| Small-pox | 536 |
| Sore Throat & Quinsey | 36 |
| Spasm | 59 |
| Stone & Gravel | 13 |
| Stricture | 14 |
| Thrush | 93 |
| Tumor | 47 |
| Venereal | 8 |
| Worms | 17 |
| Unknown Causes | 1139 |
| Casualties, as under | 439 |

| *Casualties.* | |
|---|---|
| Drowned | 104 |
| Died by Visitation of God | 53 |
| Excessive Drinking | 18 |
| Found dead | 23 |
| Killed by various Accidents | 192 |
| Murdered | 2 |
| Poisoned | 6 |
| Suicides | 41 |

Buried { Males .... 9,202; Females . 9,027 } Total, 18229.

*Of the number buried were,*

| | | | |
|---|---|---|---|
| Under 2 years of age | 4157 | 40 & under 50, | 1948 |
| 2 & under 5 yrs. | 1634 | 50 & under 60, | 1866 |
| 5 & under 10 | 783 | 60 & under 70, | 1849 |
| 10 & under 20, | 673 | 70 & under 80, | 1573 |
| 20 & under 30, | 1315 | 80 & under 90, | 685 |
| 30 & under 40, | 1651 | 90 & under 100, | 94 |
| | | 107 | 1 |

Decrease in the burials reported this year, 3186.

